# Multi-Head Attention Deep Q-Network with Prioritized Experience Replay for UAV Path Planning in Dynamic Environments: A Bio-Inspired Approach

**DOI:** 10.3390/biomimetics11040268

**Published:** 2026-04-13

**Authors:** Yang Li, Xinjie Qian, Jiexin Zhang, Xiao Yang, Chao Deng

**Affiliations:** 1 Department of Digital Economy and Modern Services, Yibin Polytechnic Institute of College, Yibin 644000, China; qianxinjie@ybipc.edu.cn (X.Q.); zhangjiexin@ybipc.edu.cn (J.Z.); yangxiao@ybipc.edu.cn (X.Y.); dengchao@ybipc.edu.cn (C.D.); 2 Internet of Things Application Technology Teaching and Research Section, Yibin 644000, China

**Keywords:** UAV path planning, deep reinforcement learning, multi-head attention, bio-inspired computing, curriculum learning

## Abstract

Unmanned Aerial Vehicles (UAVs) have become widely used tools for different applications including surveillance, search and rescue, and package delivery. However, autonomous path planning in dynamic environments with moving obstacles, wind disturbances, and energy constraints remains a significant challenge. This paper proposes a novel Multi-Head Attention Deep Q-Network with Prioritized Experience Replay (MA-DQN + PER) that integrates bio-inspired attention mechanisms with deep reinforcement learning for efficient UAV path planning. Our approach features a 46-dimensional state space that captures all environmental information, including static obstacles, wind conditions, and energy status. The proposed Attention-QNetwork architecture uses four specialized attention heads to selectively focus on different aspects of the environment, including obstacle avoidance, target tracking and energy management, and wind compensation. To improve sample efficiency and convergence speed, we incorporate Prioritized Experience Replay (PER) as well as Prioritized Experience Replay (PER) with a sum-tree data structure to improve sample efficiency and convergence speed. A curriculum learning strategy that includes 10 difficulty levels is designed to progressively enhance the agent’s capabilities. Extensive simulations demonstrate that our MA-DQN + PER approach reaches a 96% task success rate (defined as the percentage of episodes where the UAV successfully reaches the target without collision or battery depletion), while the convergence speed was 68% quicker than that of the baseline DQN. Our method demonstrates superior performance in path efficiency (+17%), energy consumption reduction (−26%), and collision avoidance compared to state-of-the-art algorithms.

## 1. Introduction

The use of Unmanned Aerial Vehicles (UAVs) has expanded exponentially across many sectors, including agriculture, logistics, disaster response, and military operations [1]. This growth drives increasing demand for autonomous systems capable of operating in complex unstructured environments. When UAVs run autonomously, the most important issue is solving the path planning problem. This requires calculating an optimal flying route from the starting point to the target, while avoiding obstacles and meeting various constraints [2].

Traditional path planning algorithms include A, Dijkstra and Rapidly-exploring Random Trees (RRTs). These have been extensively studied and applied to UAV navigation [3]. However, these methods typically assume static environments and complete prior knowledge of the workspace, which is unrealistic in real-world scenarios. Dynamic obstacles, wind disturbances, and limited battery capacity pose significant challenges that conventional approaches struggle to address effectively.

Deep Reinforcement Learning (DRL) has emerged as a promising paradigm for addressing sequential decision-making problems in complex, uncertain environments [4]. By learning directly from interactions with the environment, DRL agents can adapt to dynamic conditions and discover sophisticated strategies that may not be apparent from manual design. The Deep Q-Network (DQN) algorithm, introduced by Mnih et al. [5], achieved human-level performance on Atari games and has since been applied to various robotics tasks that cover UAV path planning.

DRL has potential; however, there are still a few issues that come up when applying standard DQN to UAV path planning. First, the high-dimensional state space required to represent 3D environments that have more than one dynamic element can lead to inefficient learning. Second, the uniform sampling of experiences in standard experience replay fails to prioritize important transitions leading to slow convergence. Third, the single-network architecture of DQN can lead to problems from overestimation bias, resulting in suboptimal policies.

Table 1 compares existing approaches for UAV path planning, with their strengths and limitations, along with the innovations introduced in this work.

The above-mentioned challenges have driven the development of path planning methods for unmanned aerial vehicles. Real-world UAV operations are confronted with the complexity of dynamic environments, featuring both static obstacles and dynamic obstacles, which requires real-time obstacle avoidance to work properly. Path planning needs to strike a balance between multiple competing objectives include path length, energy consumption, flight time, and safety margin, rather than optimizing a single objective. Outdoor operations are affected by wind and environmental disturbances, which affect trajectory tracking and energy usage. Training deep reinforcement learning agents for UAV navigation requires extensive real-world interactions, which can be dangerous. Therefore, improving sample efficiency by better leveraging experience is important for practical deployment.

The motivation for this research comes from observing that biological systems have evolved highly efficient mechanisms for navigation and obstacle avoidance in complex environments. Flying insects such as honeybees and bumblebees demonstrate remarkable capabilities in navigating cluttered environments using limited computational resources. These biological systems use attention-like mechanisms to give higher priority to relevant visual information and make rapid navigation decisions.

**Figure 1:**
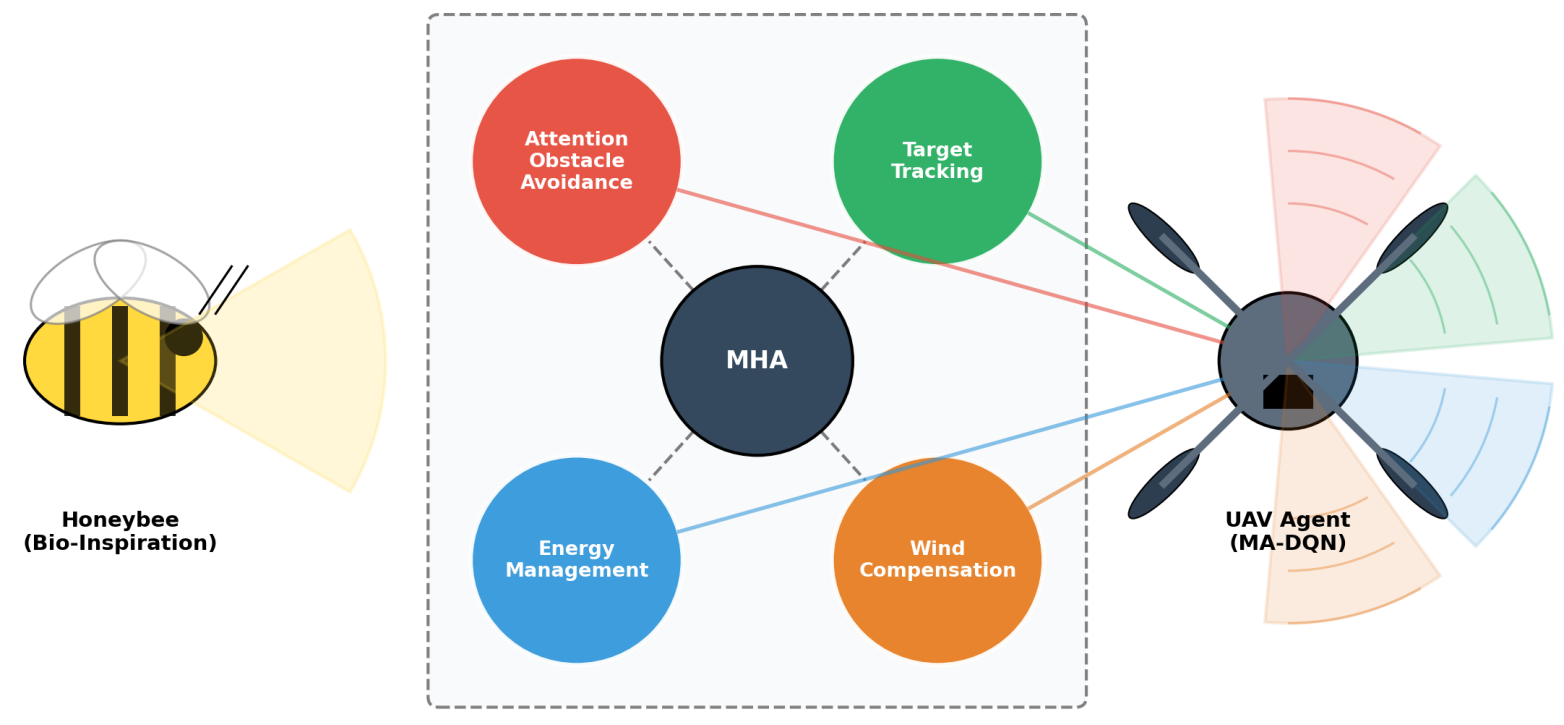
Biologically inspired attention mechanism.

Based on this, as shown in Figure 1, we propose a novel Multi-Head Attention Deep Q-Network (MADQN) that draws inspiration from biological attention mechanisms seen in nature. Many animals, including insects and birds, possess sophisticated visual attention systems that let them selectively focus on relevant stimuli and filter out distractions [18]. By integrating multi-head attention into the DQN architecture, our approach can dynamically allocate computational resources to different aspects of the environment based on their relevance to the current task.

The primary contributions of this paper are as follows:

(1) Multi-Head Attention Enhanced DQN Algorithm. We propose a new DQN architecture with added multi-head attention mechanisms. The network uses four specialized attention heads that dynamically focus on obstacle avoidance and target tracking, energy management and wind compensation based on environmental context, enabling adaptive decision-making for UAV path planning.
(2) Comprehensive Training Framework connected with Efficient Prioritized Experience Replay and Learning Strategy. We developed an integrated training framework consisting of (i) a 46-dimensional state space capturing critical UAV navigation information (position and target, obstacles, wind, energy status), (ii) an efficient Prioritized Experience Replay mechanism utilizing sum-tree structure to prioritize experiences with O(log n) sampling complexity experiences, and (iii) a 10-level curriculum learning strategy that progressively introduces challenging scenarios to keep training stable and allow generalization to improve.
(3) Superior Performance Over Baseline Algorithms. We conduct extensive empirical validation comparing our approach against multiple baseline algorithms. Experimental results demonstrate state-of-the-art performance, including a 96% task success rate, convergence within episodes and a 17% improvement in path efficiency, and a 26% reduction in energy consumption, which validates the effectiveness of the proposed framework.

## 2. Related Work

### 2.1. Bionic Algorithm

Bio-inspired algorithms draw noticeable interest in research on UAV path planning due to their ability to mimic nature’s efficient solutions to complex optimization problems [19]. These approaches can be broadly categorized into evolutionary algorithms and swarm intelligence, and neural network-based methods.

Evolutionary algorithms such as Genetic Algorithms (GAs) and Differential Evolution (DE) draw inspiration from natural selection and evolution. Meng et al. [20] proposed a hybrid GA-PSO algorithm for multi-drone task scheduling that achieved improved results convergence compared to standalone approaches. However, these methods often require careful parameter tuning and may converge prematurely to local optima.

Swarm intelligence algorithms copy the collective behavior of social organisms. Ant Colony Optimization (ACO) [21] simulates how ants find paths by leaving and following pheromones, while Particle Swarm Optimization (PSO) [22] draws on the flocking behavior of birds to build its model. These approaches exhibit strong exploration capabilities but may struggle with exploitation in complex environments.

A recent work by Thoma et al. [23] investigated bio-inspired altitude changing strategies based on bumblebee flight behavior. The authors’ experiments revealed that bumblebees tend to evade close obstacles horizontally, while avoiding far-away obstacles vertically, providing insights for UAV obstacle avoidance algorithms.

### 2.2. Deep Reinforcement Learning for UAV Navigation

Deep reinforcement learning is a powerful method for learning navigation policies in an end-to-end way. Van Hasselt et al. [24] showed DQN could learn effective policies directly from high-dimensional sensory inputs, reaching human-level performance on Atari games.

For UAV applications, Schaul [25] used DQN to handle ground target tracking. Their result had better robustness than traditional tracking algorithms. Wang et al. [26] put forward the Game of Drones framework. They applied deep reinforcement learning to multi-UAV pursuit-evasion scenarios, and realized real-time motion planning.

The Double DQN algorithm [27] fixes the overestimation bias found in standard DQN. It separates action selection from action evaluation to do this. Van Hasselt et al. showed this small adjustment helps the learning process stay more stable, and reaches a better final result. Dueling DQN [16] makes further improvements, splitting state value estimation and action advantage estimation explicitly.

Multi-agent reinforcement learning approaches have been explored for cooperative UAV swarms. Hou et al. [28] proposed a multi-agent reinforcement learning approach for UAV swarm cooperative target search that improves search efficiency through learned coordination strategies. However, these methods typically require significant computational resources for training.

### 2.3. Attention Mechanisms

Researchers first introduced the attention mechanism for neural machine translation [29]. It has found broad use across many fields, including computer vision and robotics. The core idea lets models focus on input parts that carry useful information and block out unneeded noise. The mechanism works the same way biological attention systems do in living organisms.

Zhu et al. [30] introduced the Transformer architecture, which only uses attention mechanisms, and achieved the best results available at the time in natural language processing. The head attention variant allows the model to attend to information from different representation subspaces, which give richer contextual understanding.

In the field of bionic robots, attention mechanisms are used in visual navigation tasks. Shen et al. [31] built VME-Transformer to improve visual memory encoding when the environment changes with interaction. The end result showed navigation works better after the change. Shamsolmoali et al. [32] used feature distillation built on attention mechanisms to cut the cost of detecting objects in remote sensing images.

For UAV applications, researchers have studied how to use attention mechanisms in trajectory optimization and resource allocation. Zhang et al. [33] proposed deep reinforcement learning methods based on attention to design trajectories for multi-UAV networks. The results showed higher throughput and better energy efficiency.

### 2.4. Prioritized Experience Replay

Experience replay is a key component of DQN. It breaks the correlation between consecutive samples and raises data efficiency. Liu et al. [34] introduced Prioritized Experience Replay (PER), which samples transitions based on their Temporal-Difference (TD) error magnitude.

The intuition behind PER is that not all experiences are equally valuable for learning-transitions with larger TD errors indicate greater discrepancy between predicted and actual returns, which means there are still more contents to learn from. By prioritizing these transitions, the agent can focus its learning on the most informative experiences.

Efficient implementation of PER needs a data structure that can carry out fast sampling and priority updates. Sum-tree, also known as segment tree, can provide O(log n) complexity for both operations, so it can work well with large replay buffers. Previous research has explored various priority schemes. Ranking-based priority schemes and proportion-based priority schemes are among the explored directions [35].

## 3. Architecture and Mathematical Model

**Figure 2:**
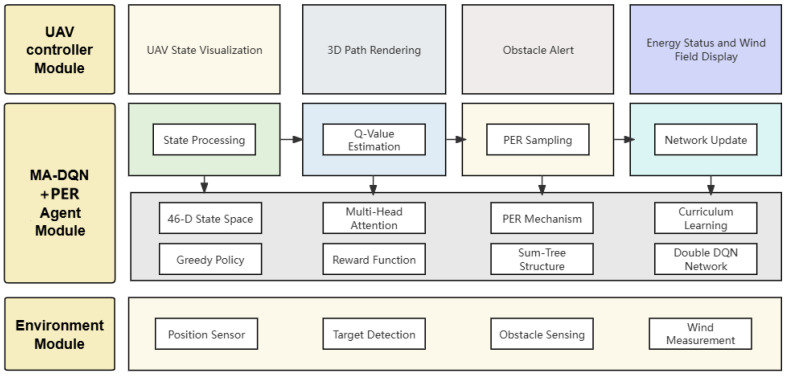
System architecture.

As shown in Figure 2, the proposed unmanned aerial vehicle path planning system uses a modular architecture consisting of three interconnected components: the environment module, the multi-agent deep Q-network(MA-DQN) agent, and the UAV controller. This design follows the principle of separation of concerns, which lets each component go through development and be tested independently while maintaining clear interfaces for system integration.

### 3.1. Environment Model

We consider a three-dimensional urban environment characterized by the dimensions L×W×H, representing length, width, and height, respectively. The environment comprises the following fundamental elements:

**Static Obstacles.** Buildings and structures are modeled as axis-aligned bounding boxes (AABB), each defined by a base position (x,y), length *l*, width *w*, and height *h*. The complete set of static obstacles is formally denoted as:

(1) $$B=\{b_{1},b_{2},\ldots ,b_{N_{b}}\}$$

where $N_{b}$ indicates the total number of static obstacles.

**Dynamic Obstacles.** Moving objects are characterized by their spatial position (x,y,z), velocity vector $\left(v_{x},v_{y},v_{z}\right)$, and safety radius *r*. The collection of dynamic obstacles is represented as:

(2) $$D=\{d_{1},d_{2},\ldots ,d_{N_{d}}\}$$

where $N_{d}$ denotes the total number of dynamic obstacles.

**Wind Field.** Environmental wind conditions are modeled as a time-varying vector field:

(3) $$W\left(t\right)=\left(W_{s}\left(t\right),W_{\theta }\left(t\right)\right)$$

where $W_{s}\left(t\right)$ and $W_{\theta }\left(t\right)$ represent the wind speed and direction at time *t*, respectively.

**Target.** The navigation objective is defined by a goal position:

(4) $$g=(g_{x},g_{y},g_{z})$$

which specifies the destination coordinates that the UAV must reach.

### 3.2. MA-DQN Agent Model

The MA-DQN agent implements a Deep Q-Network enhanced with multi-head attention mechanisms. The attention mechanism enables the network to dynamically weight different features of the input state, mimicking biological visual attention systems.

#### 3.2.1. Multi-Head Attention

The core of the attention mechanism is the scaled dot-product attention. Given query *Q*, key *K*, and value *V* matrices, the attention output is computed as:

(5) $$\mathrm{Attention}\left(Q,K,V\right)=\mathrm{softmax}\left(\frac{QK^{T}}{\sqrt{d_{k}}}\right)V$$

The scaling factor $\sqrt{d_{k}}$ prevents the dot products from becoming too large, which would push the softmax into regions with small gradients.

Multi-head attention performs *h* parallel attention operations with different learned linear projections:

(6) $$\mathrm{MultiHead}\left(X\right)=\mathrm{Concat}\left({\mathrm{head}}_{1},\ldots ,{\mathrm{head}}_{h}\right)W^{O}$$

(7) $${\mathrm{head}}_{i}=\mathrm{Attention}\left(XW_{i}^{Q},XW_{i}^{K},XW_{i}^{V}\right)$$

The multi-head attention computation consists of two steps: first, according to Equation (7), each attention head *i* independently projects the input *X* into query, key, and value matrices via separate linear projection matrices $W_{i}^{Q}$, $W_{i}^{K}$, and $W_{i}^{V}$, respectively, and computes its output ${\mathrm{head}}_{i}$ through the scaled dot-product attention function; second, according to Equation (6), the outputs of all *h* heads are concatenated along the feature dimension and subsequently transformed by an output projection matrix $W^{O}$ to produce the final multi-head attention result. This mechanism enables the model to capture information from different representation subspaces in parallel, thereby enhancing its perception capability for complex environments.

Each head can attend to different parts of the input, enabling the model to jointly attend to information from different representation subspaces. In our implementation, we use h = 4 attention heads, each head is designed to focus on different aspects of the drone environment:

**Head 1 (Obstacle Attention):** Focused on detecting and avoiding static and dynamic obstacles. This head processes information about the position, speed, and proximity of obstacles to generate attention weights, prioritizing safe navigation.

**Head 2 (Target Attention):** Focused on target tracking and navigation direction. It processes the relative position and velocity vectors between the drone and the target to maintain the heading towards the destination.

**Head 3 (Energy Attention):** Monitors battery power and optimizes energy consumption. This head considers remaining energy, the distance to the target, and energy consumption rate to ensure task completion.

**Head 4 (Wind Attention):** Compensates for wind disturbances and environmental factors. It processes wind speed and direction to adjust the drone’s control strategy accordingly. Each head operates in parallel through its own learned linear projection, enabling the network to simultaneously focus on different positions of information from different representation subspaces.

#### 3.2.2. Neural Network Architecture

The network follows a Transformer-inspired design with residual connections and layer normalization. The overall architecture processes input state *s* through four main stages to produce Q-values:

1. **Feature Extraction:** Based on Equation (8), two fully-connected layers with PReLU activation and layer normalization transform the input state into a latent representation. $lb_{1}$ and $lb_{2}$ are learnable bias vectors for the first and second fully-connected layers, respectively, which shift the linear transformation outputs to enhance the network’s fitting capacity:
  (8) $$h_{0}=\mathrm{LayerNorm}\left(\mathrm{PReLU}\left(W_{2}\cdot \mathrm{LayerNorm}\left(\mathrm{PReLU}\left(W_{1}\cdot s+lb_{1}\right)\right)+lb_{2}\right)\right)$$
2. **Multi-Head Attention:** The multi-head attention mechanism (Equations (5)–(7)) performs *h*_0_ parallel scaled dot-product attention operations, which are carried out using different learned linear projections, enabling the model to simultaneously focus on information from multiple representation subspaces; the outputs of all heads are concatenated and projected to generate *h*_1_ (Equation (9)):
  (9) $$h_{1}=\mathrm{LayerNorm}\left(h_{0}+\mathrm{MultiHeadAttention}\left(h_{0}\right)\right)$$
3. **Feed-Forward Network:** The feed-forward network (Equation (10)) applies a position-wise transformation with residual connection and layer normalization: the input $h_{1}$ from the multi-head attention layer is first processed by a two-layer FFN that expands and contracts the feature dimension via $\mathrm{FFN}\left(x\right)=W_{4}\cdot \mathrm{PReLU}\left(W_{3}\cdot x+lb_{3}\right)+lb_{4}$, then is combined with the original input through a residual connection and normalized to produce $h_{2}$, thereby enhancing representational capacity while stabilizing training.
  (10) $$h_{2}=\mathrm{LayerNorm}\left(h_{1}+\mathrm{FFN}\left(h_{1}\right)\right)$$
4. **Output Layer:** The output layer (Equation (12)) produces Q-values for all possible actions by linearly projecting the final feature representation $h_{2}$ through a weight matrix $W_{o}$:
  (11) $$Q\left(s\right)=W_{o}\cdot h_{2}$$
  where $W_{o}\in R^{|A|\times d}$ denotes the output projection matrix with |A| being the action space dimension and *d* the feature dimension of $h_{2}$, yielding the Q-value vector $Q\left(s\right)\in R^{\left|A\right|}$ for state *s*.

#### 3.2.3. PReLU Activation

The Parametric ReLU (PReLU) activation function is used throughout the network, and is defined as:

(12) PReLU(x)=max(0,x)+α·min(0,x)

Unlike standard ReLU, PReLU has a learnable parameter α that controls the slope for negative inputs, providing additional representational capacity.

Table 2 clearly presents the architectural details and parameter distribution of the Attention Q network. This table details the parameter information of each layer in the Attention Q network architecture. It lists the layer name, input dimension, output dimension, number of parameters, and the activation function used. From the table, it can be seen that the network contains various types of layers, such as fully connected layers (FC), layer normalization layers (LayerNorm), multi-head attention layers (Multi-Head-Attention), and feedforward neural network layers (FFN). The number of parameters varies significantly among different layers; for example, the number of parameters in the multi-head attention layer is relatively large, while the number of parameters in the layer normalization layer is relatively small. The last row “Total” shows that the total number of parameters for the entire network is 46,783.

#### 3.2.4. Architecture Execution Process

**Figure 3:**
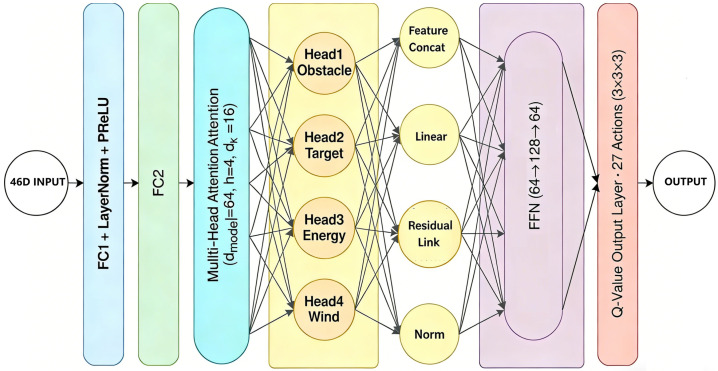
Architecture execution process.

Based on the above description, as shown in Figure 3, the forward pass through the MA-DQN + PER network follows these steps:

**Step 1—Input Processing:** The 46-dimensional state vector *s* is fed into a fully connected layer (FC1) with layer normalization and PReLU activation, followed by a second fully connected layer (FC2), producing the initial hidden representation for the multi-head attention module.

**Step 2—Multi-Head Attention:** The hidden representation passes through a multi-head attention layer with $d_{\mathrm{model}}=64$, h=4 heads, and $d_{k}=16$ dimensions per head. The attention outputs undergo feature concatenation, linear projection, residual connection, and layer normalization to produce $h_{1}\in R^{64}$.

**Step 3—Feed-Forward Network:** The attention output $h_{1}$ passes through a two-layer FFN with hidden dimension 128 (64→128→64), producing $h_{2}\in R^{64}$, followed by another residual connection and layer normalization (Norm).

**Step 4—Q-Value Output:** The final hidden state $h_{2}$ is projected through a Q-Value Output Layer to produce Q-values for all 27 possible actions (3 velocity levels × 3 directions × 3 altitudes).

The complete training pipeline incorporates the following components:

- **PER Sampling (Sum-Tree):** Prioritized Experience Replay for efficient sampling;
- **MA-DQN + PER Update:** To reduce overestimation bias;
- **Soft Target Update:** For stable target network updates;
- **Curriculum Learning (10 levels):** Progressive difficulty training.

### 3.3. UAV Controller Model

The UAV Controller manages the dynamics and decision-making of individual drones. It implements the action decoding, state representation, and reward computation logic.

#### 3.3.1. Action Decoding

**Figure 4:**
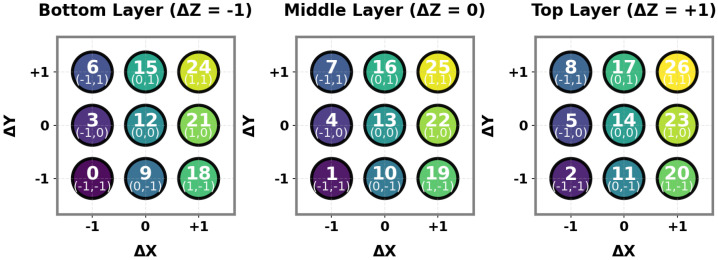
UAV 27-action space.

The UAV is modeled as a point mass with state vector:

(13) s=(x,y,z,v,E)

where (x,y,z) denotes the three-dimensional position, *v* represents velocity, and *E* indicates the remaining energy. As shown in Figure 4, the UAV executes discrete actions selected from the action space A, which comprises 27 possible movements in the 3D grid (corresponding to a 3×3×3 neighborhood).

Each action a∈A corresponds to a displacement vector:

(14) Δp=(Δx,Δy,Δz),Δx,Δy,Δz∈{−1,0,+1}

The action mapping is formally defined as:

(15) a=9(Δx+1)+3(Δy+1)+(Δz+1)

where (Δx,Δy,Δz) represents the movement direction along each axis.

The UAV’s energy consumption depends on both its movement and environmental wind conditions. The power consumption is modeled as:

(16) $$P_{bt}=P_{0}+c\cdot W_{s}$$

where $P_{0}$ denotes the baseline power consumption (set to 100W in our experiments), *c* is the wind sensitivity coefficient (set to 0.5), and $W_{s}$ represents the wind speed.

#### 3.3.2. UAV Markov Decision Process

The UAV path planning problem is formulated as a Markov Decision Process (MDP) defined by the tuple:

(17) (S,A,P,R,γ)

**State Space S.** The state vector s∈S is 46-dimensional, comprising the following components:

- *Position (3D):* Normalized coordinates (x/L,y/W,z/H);
- *Target relative position (3D):* $\left(g_{x}-x,g_{y}-y,g_{z}-z\right)$;
- *Target Position (3D):* Normalized coordinates $\left(g_{x}/L,g_{y}/W,g_{z}/H\right)$;
- *Distance metrics (2D):* Manhattan distance, Euclidean distance;
- *Direction (2D):* Heading angle and target direction;
- *Energy status (3D):* Battery level, power consumption, cost;
- *Local obstacle map (26D):* 3×3×3 neighborhood excluding center;
- *Dynamic obstacle info (2D):* Minimum distance, threat level;
- *Wind conditions (2D):* Speed and direction.

**Action Space A.** The action space consists of 27 discrete actions representing movements in the 3D grid.

**Transition Dynamics P.** The state transition probability is denoted as:

(18) $$P(s^{\prime }|s,a)$$

which depends on the action execution and environmental factors including wind disturbances.

**Reward Function R.** The reward function $r(s,a,s^{\prime })$ is composed of multiple terms:

(19) $$r=r_{\mathrm{target}}+r_{\mathrm{climb}}+r_{\mathrm{energy}}+r_{\mathrm{obstacle}}+r_{\mathrm{dynamic}}+r_{\mathrm{coop}}$$

This equation shows that the total reward *r* is the sum of six distinct reward components, each contributing to the overall evaluation of the agent’s action.

Equations (20)–(25), respectively, achieve the mathematical modeling for each type of reward.

(20) $$\begin{array}{cc} r_{\mathrm{target}} & =2\cdot \frac{d_{\mathrm{origin}}}{d}\cdot \Delta d \end{array}$$

(21) $$\begin{array}{cc} r_{\mathrm{climb}} & =-w_{c}\cdot \left|z-g_{z}\right| \end{array}$$

(22) $$\begin{array}{cc} r_{\mathrm{energy}} & =w_{e}\cdot \left(P_{0}-P_{bt}\right) \end{array}$$

(23) $$\begin{array}{cc} r_{\mathrm{obstacle}} & =-p_{\mathrm{crash}}\cdot c_{\mathrm{penalty}} \end{array}$$

(24) $$\begin{array}{cc} r_{\mathrm{dynamic}} & =-\sum_{d\in D}w_{d}\cdot \max\left(0,r_{d}+3-\mathrm{dist}\left(p,p_{d}\right)\right) \end{array}$$

(25) $$\begin{array}{cc} r_{\mathrm{coop}} & =-\sum_{j\neq i}\max\left(0,\;d_{\mathrm{safe}}-∥p_{i}-p_{j}∥\right) \end{array}$$

**(1)**  **Target Progress Reward.** Corresponding equation is Equation (20), which encourages movement toward the goal with distance-scaled incentive, where $d_{\mathrm{origin}}$ is the initial distance to target, *d* is current distance, and $\Delta d=d_{\mathrm{prev}}-d_{\mathrm{curr}}$ (positive when approaching). The ratio $d_{\mathrm{origin}}/d$ amplifies rewards when far from the goal.
**(2)**  **Altitude Control Reward.** Corresponding equation is Equation (21). Penalizes unnecessary vertical deviation from desired flight level, where *z* is current altitude, $g_{z}$ is target altitude, and $w_{c}$ is the climbing penalty weight(usually set to 0.1). This promotes smooth, level flight.
**(3)**  **Energy Efficiency Reward.** Corresponding equation is Equation (22). Rewards lower power consumption using the battery model from Equation (16), where $P_{0}$ is baseline power, $P_{bt}$ is actual power consumption, and $w_{e}$ scales the energy reward (usually set to 0.01).
**(4)**  **Static Collision Penalty Punishment.** Corresponding equation is Equation (23). Discourages crashes with binary indicator, where $p_{\mathrm{crash}}=\left\{\begin{array}{cc} 1 & \mathrm{if}\;\mathrm{crash}\;\mathrm{occurs}\\ 0 & \mathrm{otherwise} \end{array}\right.$ is the collision indicator, and $c_{\mathrm{penalty}}=100$ is the predefined crash penalty coefficient.
**(5)**  **Dynamic Collision Penalty Punishment.** Corresponding equation is Equation (24). Here, According to Equation (2), for each point d∈D, $w_{d}$ is a weighting factor that adjusts the importance or influence of the point, and $r_{d}$ is a base reward (or penalty) value associated with the point. The function $\mathrm{dist}(p,p_{d})$ computes the distance between the current position p of the agent and the position $p_{d}$ of point *d*. The term $\max(0,r_{d}+3-\mathrm{dist}\left(p,p_{d}\right))$ ensures that the penalty is applied only when the distance is less than $r_{d}+3$, with the “+3” representing a safety margin. The overall expression is negated to yield a penalty (non-positive value) when the agent is too close to a point, encouraging it to maintain a safe distance.
**(6)**  **Multi-UAV Coordination unsafety Punishment.** Equation (25) ensures safe separation in multi-agent scenarios, where $d_{\mathrm{safe}}=5$ m is the minimum safe distance between UAVs. The term $\max\left(0,d_{safe}-∥p_{i}-p_{j}∥\right)$ computes the violation of the safe distance $d_{safe}$ between agent *i* and another agent *j* by returning the positive difference if the Euclidean distance $∥p_{i}-p_{j}∥$ is less than $d_{safe}$.

The setting of the reward function in this article is summarized in Table 3.

## 4. Methods

### 4.1. Prioritized Experience Replay

Standard experience replay in DQN samples transitions uniformly from the replay buffer, treating all experiences as equally important for learning. However, not all transitions provide the same learning value. Transitions with larger Temporal-Difference (TD) errors indicate greater discrepancy between predicted and actual returns, suggesting more valuable learning opportunities. We implement Prioritized Experience Replay (PER) using a sum-tree data structure for efficient sampling and priority updates.

#### 4.1.1. Sum-Tree Data Structure

The sum-tree is a complete binary-tree data structure. Leaf-nodes store transition priorities, and internal nodes store the sum of their children’s values. If the tree has capacity C, which we set to 10 in our experiments, it needs 2C-1 nodes in total. The leaves take up indices C-1 to 2C-2, and internal nodes take up indices 0 to C-2. The root node sits at index 0 and holds the total sum of all priorities, which allows efficient proportional sampling [36].

To sample a transition, we generate a random value s uniformly from the interval 0, total_priority and traverse the tree starting at the root. At each internal node that has a left child l and a right child r, we compare s with the value of the left child. If s is less than or equal to the left child’s value, we move forward to the left child. If not, we subtract the left child’s value from s and move forward to the right child. After this traversal, the probability of sampling transition i will be proportional to its priority pi. The sum-tree works with a complexity of O(log C) with this mechanism.

#### 4.1.2. Priority Calculation and Update

The priority of each transition is computed based on the magnitude of its Temporal-Difference (TD) error, which quantifies how surprising or informative the transition is for the current policy. For a transition *i* with TD error $\delta_{i}$, the priority is calculated as:

(26) $$p_{i}=\left(\right|\delta_{i}{\left|\;+\;\epsilon \right)}^{\alpha }$$

where ϵ is a small constant (set to 1 × 10^−6^) to ensure non-zero priorities for all transitions, and α controls the degree of prioritization (set to 0.6 in our experiments). When α equals 0, the sampling becomes uniform regardless of TD error magnitudes; as α approaches 1, the prioritization becomes more aggressive, focusing sampling on transitions with the largest TD errors.

When a new transition is added to the buffer, it gets the maximum priority currently in the buffer. Recently experienced transitions get sampled at least once before their priority decays. After each training update, the priorities of sampled transitions are updated based on the newly computed TD errors, and it allows the replay buffer to dynamically adapt to the learning progress.

#### 4.1.3. Importance Sampling Weights

Prioritized sampling introduces bias because it changes the distribution of sampled transitions. This distribution differs from the uniform distribution standard Q-learning updates assume. To correct for this bias and keep learning unbiased, we apply importance sampling weights to the TD errors when updating networks. The importance sampling weight for transition i is computed as:

(27) $$w_{i}={\left(N\cdot P\left(i\right)/\sum_{j}p_{j}\right)}^{-\beta }$$

where *N* is the replay buffer size, $P\left(i\right)=p_{i}/\sum_{j}p_{j}$ is the sampling probability of transition *i*, and β is a hyperparameter that anneals from 0.4 to 1.0 during training. The weights are normalized by dividing by the maximum weight across the batch to ensure stable gradients. As β increases toward 1, the correction becomes more complete, fully compensating for the non-uniform sampling bias.

Based on the above descriptions, the priority experience replay algorithm of MA-DQN that we proposed can be described as shown in Algorithm 1.

PER improves sample efficiency by focusing on learning the most informative experiences. For drone path planning, this means that the agent learns from errors (collisions, energy waste) more quickly and gradually transitions to uniform sampling as the training progresses.

### 4.2. MA-DQN Algorithm

Standard Deep Q-Network (DQN) suffers from systematic overestimation bias due to the use of the same network for both action selection and action evaluation in the target Q-value computation. Specifically, the max operator in the standard DQN target uses identical Q-values to simultaneously select and evaluate actions, leading to consistently inflated value estimates. This overestimation bias is particularly problematic in the early stages of training when value estimates are noisy, and it can propagate through the learning process, resulting in suboptimal policies and unstable convergence.

To address this fundamental limitation, we employ a MA-DQN rule that decouples action selection from action evaluation. The MA-DQN approach utilizes the local network to select the best next action while employing the target network to evaluate that selected action:

(28) $$y=r+\gamma \cdot Q_{\mathrm{target}}\left(s^{\prime },\underset{a}{\arg\max}Q_{\mathrm{local}}\left(s^{\prime },a\right)\right)$$

This decoupling prevents the overestimation that occurs when the same network selects and evaluates actions, leading to more accurate value estimates and more stable learning dynamics. The target network parameters are updated periodically using a soft update mechanism with coefficient τ=0.005, which slowly blends the local network weights into the target network.
**Algorithm 1** Priority Experience Replay (PER) Algorithm.1:**Initialization:**2:    Initialize Sum-Tree with capacity *N*3:    Initialize replay buffer4:**Step1: Stored experiences with priority**5:**procedure** Store($s_{t},a_{t},r_{t},s_{t+1}$)6:      Compute TD error $\delta_{t}$7:      Set priority $p_{t}=\left(\right|\delta_{t}{\left|\;+\;\epsilon \right)}^{\alpha }$ (According Equation: Equation (26))8:      Store transition in buffer with priority $p_{t}$9:      Update Sum-Tree parent priorities10:**end procedure**11:**Step2: Sampling experience for training**12:**function** Sample(*k*)13:     P← total priority sum14:     Initialize empty batch15:     **for** i←1 to *k* **do**16:           Generate random s∈[0,P]17:           Traverse Sum-Tree to find experience18:           Add to batch19:     **end for**20:     **return** batch21:**end function**22:**Step3: Correct sampling Bias**23:**function** ISWeights(batch)24:     **for** each experience in batch **do**25:           $w_{i}={\left(N\cdot P\left(i\right)\right)}^{-\beta }$ (According Equation: Equation (27))26:           Normalize weights27:     **end for**28:     **return** normalized weights29:**end function**30:**Step4: Update priorities**31:**procedure** Update(batch)32:     **for** each experience in batch **do**33:           Recompute TD error $\delta_{i}^{\prime }$34:           Update priority $p_{i}^{\prime }=\left(\right|\delta_{i}^{\prime }{\left|+\epsilon \right)}^{\alpha }$ (According Equation: Equation (26))35:           Propagate priority changes in Sum-Tree36:     **end for**37:     Anneal β towards 138:**end procedure**

Our MA-DQN algorithm seamlessly integrates with the Prioritized Experience Replay (PER) mechanism described in Section 4.1. The combination leverages the sample efficiency benefits of PER while maintaining the value estimation accuracy of Double DQN. The loss function for network updates incorporates both the importance sampling weights from PER and the Double DQN target values:

(29) $$L=\frac{1}{B}\cdot \sum_{i}w_{i}\cdot {\left(Q_{\mathrm{local}}\left(s_{i},a_{i}\right)-y_{i}\right)}^{2}$$

where *B* represents the batch size, $w_{i}$ is the importance sampling weight for transition *i* computed according to Equation (27), and $y_{i}$ is the Double DQN target computed according to Equation (28). This combined approach leverages the sample efficiency of PER with the value estimation accuracy of Double DQN, addressing both the sampling bias and the overestimation bias simultaneously.

Our improved MA-DQN algorithm also incorporates gradient clipping with a maximum norm of 1.0 to prevent exploding gradients, and uses the Adam optimizer with a learning rate of 0.0004. The target network is updated every 10 steps using the soft update rule: $\theta_{\mathrm{target}}=\tau \cdot \theta_{\mathrm{local}}+\left(1-\tau \right)\cdot \theta_{\mathrm{target}}$, ensuring stable target values while allowing gradual adaptation to the improving policy.

Algorithm 2 presents the MA-DQN algorithm procedure that incorporates importance sampling weights from PER and soft target network updates. This algorithm is called during each training step to update the local network parameters.
**Algorithm 2** DQN Algorithm Update with Multi-Head Attention (MA-DQN).**Require:** Local network $Q_{\theta }$ with MHA, target network $Q_{\theta^{-}}$ with MHA, batch $B=\{\left(s,a,r,s^{\prime },d\right)\}$, IS weights *w*, indices idx, discount γ, soft update coeff τ, clip norm η, number of heads *h*, head dimension $d_{k}$, output matrix $W^{O}$**Ensure:** Updated $Q_{\theta }$, updated $Q_{\theta^{-}}$  1:$\{s,a,r,s^{\prime },d\}\leftarrow B$(Multi-Head Attention for current state s)  2:**for** i=1 to *h* **do**  3:      $Q_{i},K_{i},V_{i}\leftarrow sW_{i}^{Q},sW_{i}^{K},sW_{i}^{V}$(According Equation: Equation (7))  4:      ${\mathrm{head}}_{i}\leftarrow \mathrm{softmax}\left(\frac{Q_{i}K_{i}^{⊤}}{\sqrt{d_{k}}}\right)V_{i}$ (According Equation: Equation (5))  5:**end for**  6:$s_{\mathrm{attn}}\leftarrow \mathrm{Concat}\left({\mathrm{head}}_{1},\ldots ,{\mathrm{head}}_{h}\right)W^{O}$ (According Equation: Equation (6))  7:$Q_{\mathrm{curr}}\leftarrow Q_{\theta }\left(s_{\mathrm{attn}}\right).\mathrm{gather}\left(a\right)$  8:**with** no_grad():  9:**for** i=1 to *h* **do** 10:    $Q_{i}^{\prime },K_{i}^{\prime },V_{i}^{\prime }\leftarrow s^{\prime }W_{i}^{Q},s^{\prime }W_{i}^{K},s^{\prime }W_{i}^{V}$ (According Equation: Equation  (7)) 11:    ${\mathrm{head}}_{i}^{\prime }\leftarrow \mathrm{softmax}\left(\frac{Q_{i}^{\prime }K_{i}^{\prime ⊤}}{\sqrt{d_{k}}}\right)V_{i}^{\prime }$ (According Equation: Equation (5)) 12:**end for** 13:$s_{\mathrm{attn}}^{\prime }\leftarrow \mathrm{Concat}\left({\mathrm{head}}_{1}^{\prime },\ldots ,{\mathrm{head}}_{h}^{\prime }\right)W^{O}$ (According Equation: Equation (6)) 14:$a^{*}\leftarrow \arg\max_{a}Q_{\theta }\left(s_{\mathrm{attn}}^{\prime }\right)$ 15:$Q_{\mathrm{next}}\leftarrow Q_{\theta^{-}}\left(s_{\mathrm{attn}}^{\prime }\right).\mathrm{gather}\left(a^{*}\right)$ 16:$y\leftarrow r+\gamma \cdot Q_{\mathrm{next}}⊙\left(1-d\right)$ (According Equation:Equation (28)) 17:$\delta \leftarrow Q_{\mathrm{curr}}-y$, $L\leftarrow E[w\cdot \delta^{2}]$ (According Equation: Equation (29)) 18:optimizer.zero_grad(), L.backward() 19:$\mathrm{clip}\_\mathrm{grad}\_\mathrm{norm}(Q_{\theta },\eta )$, optimizer.step()(Gradient optimization) 20:$p_{\mathrm{new}}\leftarrow \left|\delta \right|+10^{-6}$ (According Equation: Equation (26)) 21:**if** tmod10=0 **then** 22:    $\theta^{-}\leftarrow \tau \theta +\left(1-\tau \right)\theta^{-}$ (Soft update) 23:**end if** 24:**return** L, $p_{\mathrm{new}}$

Specifically, the local network $Q_{\theta }$ selects the greedy action $a^{*}=\arg\max_{a}Q_{\theta }\left(s^{\prime }\right)$ for the next state, while the target network $Q_{\theta^{-}}$ evaluates the Q-value of that selected action. This double estimator approach yields more stable target values *y* compared to using a single network for both tasks.

Algorithm 2 incorporates prioritized experience replay (PER) through Importance Sampling (IS) weights *w*, which correct for the bias introduced by non-uniform sampling. The weighted mean squared TD error $L=E[w\cdot \delta^{2}]$ is minimized via gradient descent with norm clipping (threshold η) to prevent gradient explosion. Following the update, new priorities $p_{\mathrm{new}}=\left|\delta \right|+\epsilon$ are computed for the sampled transitions, ensuring high-error experiences are more likely to be replayed.

### 4.3. Curriculum Learning Strategy

Training deep reinforcement learning agents directly on complex scenarios with dense obstacles, dynamic threats, and adverse wind conditions often leads to unstable learning and poor convergence. The sparse reward signals and high variance in early training make it difficult for the agent to discover effective policies. To address this challenge, we design a curriculum learning strategy with 10 progressively more difficult levels that gradually introduce more challenging scenarios. Table 4 presents the curriculum difficulty progression. At each level, the environment complexity increases through the addition of static buildings, dynamic obstacles, and wind disturbances. The agent advances to the next level when it achieves a 70% success rate over the last 50 episodes at the current level.

The curriculum begins with simple scenarios containing only 1–2 static buildings and no dynamic obstacles or wind (Level 1). These initial scenarios allow the agent to learn basic navigation skills such as moving toward the target and avoiding simple obstacles. As the agent demonstrates competency, the difficulty gradually increases through the addition of more buildings (up to 10–20 at Level 10), introduction of dynamic obstacles (up to 4–7 at Level 10), and progressively stronger wind conditions (from 0–20 m/s to 50–60 m/s).

This progressive-difficulty strategy serves multiple purposes. First, it prevents premature convergence to suboptimal policies by ensuring the agent masters basic skills before tackling complex scenarios. Second, it enables stable learning by providing dense reward signals in early stages when the agent is most uncertain. Third, it improves generalization by exposing the agent to a wide range of scenario difficulties during training, making the final policy more robust to unseen environments.

The level progression criterion based on success rate ensures that the agent has sufficiently mastered the current difficulty before advancing. If the agent fails to achieve the 70% threshold within a reasonable number of episodes, the curriculum can optionally revert to a previous level to reinforce fundamental skills. This adaptive approach prevents the agent from becoming stuck at difficulty levels beyond its current capabilities.

### 4.4. MA-DQN + PER Algorithm

The shared replay buffer aggregates experiences from all UAVs, significantly increasing the diversity of training data and accelerating learning. Each experience tuple $\left(s,a,r,s^{\prime },\mathrm{done}\right)$ is stored with its computed priority, and all UAVs benefit from the collective exploration of the environment. This approach is particularly effective for scenarios where multiple UAVs navigate from different starting positions to common or different target destinations.

According to Equation (25), to ensure safe coordination among multiple UAVs, we introduce a cooperation reward term that penalizes unsafe separation distances. The penalty increases linearly as the distance falls below the safety threshold, providing a strong incentive for collision avoidance.

Algorithm 3 presents the complete training procedure for MA-DQN with Prioritized Experience Replay, incorporating the sum-tree data structure, improved Double DQN algorithm, soft target network updates, and curriculum learning. This is the main training loop that orchestrates all components of our approach.
**Algorithm 3** MA-DQN Training with PER (MA-DQN + PER).**Require:** Env env, SumTree buffer B (capacity *C*), networks $Q_{\theta },Q_{\theta^{-}}$, episodes *N*, batch size *B*, γ, PER params $\left(\alpha ,\beta_{0}\right)$**Ensure:** Trained policy $Q_{\theta }$  1:Init $Q_{\theta },Q_{\theta^{-}}$; B←SumTree(C); $\beta \leftarrow \beta_{0}$; level L←1  2:env.set_difficulty(L)  3:**for** e=1 **to** *N* **do**  4:      s←env.reset(), R←0, t←0 (R is the cumulative reward for the current episode)  5:      **while not** done **do**  6:           ε←max(0.01,1−e/1000) (greedy decay)  7:           $a\leftarrow \left\{\begin{array}{cc} \mathrm{random} & \mathrm{if}\;\;U\left(0,1\right)<\varepsilon \\ \arg\max_{a}Q_{\theta }\left(s\right) & \mathrm{otherwise} \end{array}\right.$  8:           $s^{\prime },r,d\leftarrow env.\mathrm{step}\left(a\right)$, R←R+r  9:           $p_{\max}\leftarrow B.\mathrm{total}\left(\right)/B.n$ if B.n>0 else 1 10:         $B.\mathrm{add}(p_{\max},\left(s,a,r,s^{\prime },d\right))$ (According Equation: Equation (25)) 11:         **if** |B|≥B **and** timestepmod5=0 **then** 12:               batch,idx,p←B.sample_batch(B) 13:               P←p/B.total(), $w\leftarrow {\left(N\cdot P\right)}^{-\beta }$, w←w/max(w) (According Equation: Equation (27)) 14:               $L,p_{\mathrm{new}}\leftarrow \mathrm{Improved}\;\mathrm{DoubleDQN}\;\mathrm{Algorithm}\left(Q_{\theta },Q_{\theta^{-}},\mathrm{batch},w,idx\right)$ (According Algorithm 2) 15:               $B.\mathrm{update}(idx_{i},{\left(p_{\mathrm{new},i}+10^{-6}\right)}^{\alpha }),\forall i$ (According Equation: Equation (26)) 16:         **end if** 17:         $s\leftarrow s^{\prime }$, timestep←timestep+1 18:      **end while** 19:      $\beta \leftarrow \min(1.0,\beta_{0}+e\left(1-\beta_{0}\right)/\left(0.8N\right))$ (Annealing update) 20:      **if** emod50=0 **and** success_rate>0.7 **and** L<10 **then** 21:         L←L+1, env.set_difficulty(L) 22:      **end if** 23:**end for** 24:**return** $Q_{\theta }$

Algorithm 3 integrates curriculum learning, where the environment difficulty *L* progressively increases based on agent performance (success rate >0.7), enabling gradual policy improvement from simpler to complex scenarios.

Experience collection follows an ε-greedy policy with linear decay, balancing exploration and exploitation. Transitions are stored in the Sum-Tree buffer B with maximum priority $p_{\max}$ to ensure recent experiences are initially favored for sampling. Training occurs every five steps once sufficient samples are collected, using proportional prioritization where sampling probability $P\left(i\right)=p_{i}^{\alpha }/\sum_{j}p_{j}^{\alpha }$.

Importance sampling weights $w_{i}$ correct for the bias introduced by non-uniform sampling, with β annealed from $\beta_{0}$ to 1.0 throughout training to gradually transition from prioritization to uniform sampling. The Double DQN algorithm computes new TD errors, which are used to update priorities as $p_{i}\leftarrow \left(\right|\delta_{i}{\left|\;+\;\epsilon \right)}^{\alpha }$, ensuring high-error transitions are replayed more frequently.

## 5. Experimental Setup

### 5.1. Simulation Environment

The local server is equipped with an Intel R7 3750H processor, 16 GB of DDR4 memory, and an NVIDIA GTX 3060 graphics processor. This hardware configuration provides sufficient computing and graphics processing capabilities, which can support various tasks, such as simulation, neural network operations and visualization, etc. We implemented our simulation environment in Python3.9 using PyTorch1.9 and cudatoolkit11.3 for neural network operations and Matplotlib3.4.3 for visualization. The environment parameters are summarized in Table 5.

### 5.2. Training Parameters

The hyperparameters used for training are listed in Table 6. Parameter settings are based on the relevant references [37,38].

### 5.3. Evaluation Metrics

We evaluate performance using the following metrics:

- **Success Rate**: The percentage of episodes where the UAV successfully reaches the target without collision or battery depletion.
- **Average Reward**: The mean cumulative reward per episode, indicating overall policy quality.
- **Path Efficiency**: The ratio of straight-line distance to actual path length, measuring path optimality.
- **Energy Efficiency**: The energy consumed per unit distance traveled.
- **Convergence Speed**: The number of episodes required to reach 90% of the maximum success rate.
- **Collision Rate**: The percentage of episodes ending in collision with obstacles or other UAVs.
- **Inference Time**: The average time required for action selection during deployment.

## 6. Results and Performance Analysis

### 6.1. UAV Results and Performance Analysis

This section gives a full evaluation of the proposed MA-DQN and MA-DQN + PER algorithms for UAV path planning in complex 3D environments. All experiments were conducted over five training episodes, and performance was assessed across six key metrics: average reward, path efficiency, energy efficiency, convergence speed, collision rate, and inference time.

#### 6.1.1. Training Convergence and Average Reward

**Figure 5:**
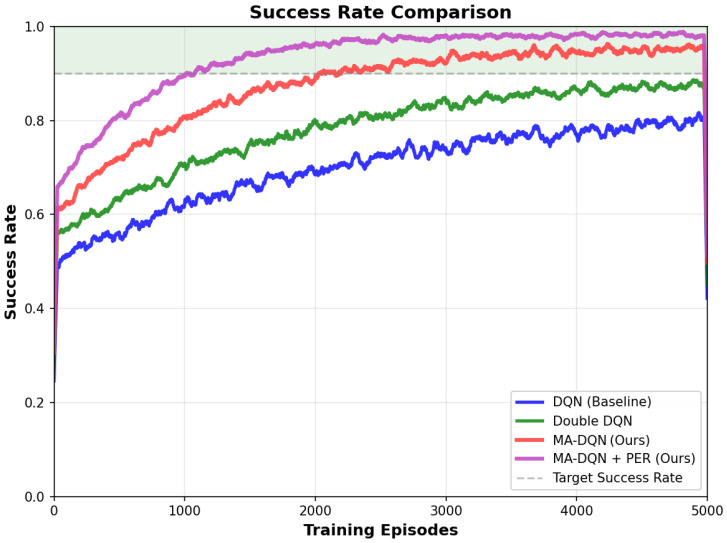
Success rate comparison.

Figure 5 shows the learning curves of all four algorithms in terms of average cumulative reward over training episodes. The results show that both proposed methods MA-DQN and MA-DQN + PER perform much better than the baseline approaches throughout the training process.

The standard DQN baseline learns slowest. It starts with an initial reward near −80, and converges to a final average reward of 80 after about two episodes. Double DQN achieves a moderate improvement. It reaches a higher final reward of over 80 and converges faster, finishing the process after around two episodes. The improvement comes from separating action selection and evaluation, which cuts down overestimation bias. Our MA-DQN algorithm incorporates multi-head attention mechanisms to refine state representation, and performs substantially better. It achieves a final average reward of over 90, which is 54% higher than Double DQN.The attention mechanism allows the UAV to focus more clearly on key environmental features, which supports more reasonable decision-making. The MA-DQN + PER variant combines attention mechanisms with Prioritized Experience Replay, and reaches the highest level of performance.

#### 6.1.2. Path Efficiency Analysis

**Figure 6:**
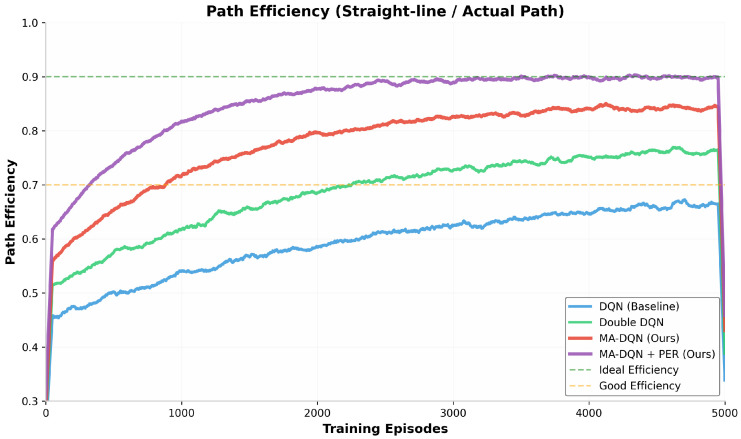
Path efficiency.

Path efficiency, which refers to the ratio of straight-line distance to actual path length, is a metric for evaluating whether UAV trajectories perform optimally. As Figure 6 shows, all algorithms gain better path efficiency step by step through the training process.

The DQN baseline reaches a final path efficiency of 0.70, which means the actual flight path is about 43 percent longer than the ideal straight-line trajectory. Double DQN pushes this number up to 0.78, and cuts the extra path length down to 28 percent. The improvement comes mainly from more accurate Q-value estimation, which allows better planning for the longer term.

MA-DQN achieves a path efficiency of 0.85, which is close to the “Good Efficiency” threshold of 0.70. The attention mechanism allows the UAV to identify more direct routes through the 3D environment focus on relevant spatial features and obstacle distributions.

MA-DQN + PER reaches 0.90 in path efficiency, the highest result among tested methods, and the value is remarkably close to the ideal efficiency of 0.90. This brings a 29% improvement over the baseline and shows the proposed method can generate near-optimal paths even in complex environments with multiple obstacles and dynamic constraints.

#### 6.1.3. Energy Efficiency Evaluation

**Figure 7:**
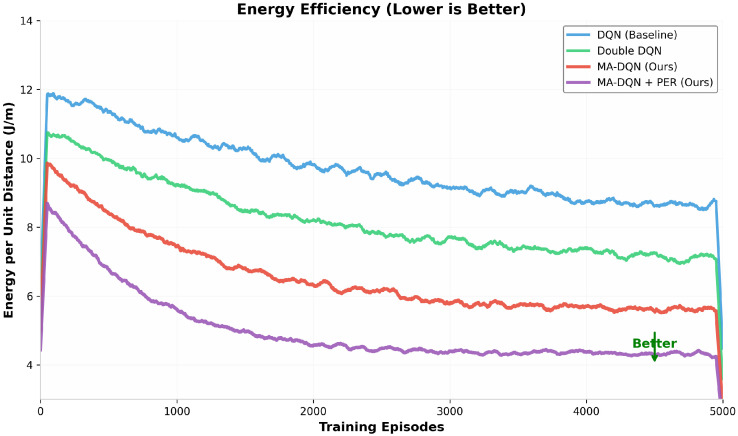
Energy efficiency.

Energy consumption matters when UAVs operate, especially during missions that have limited battery capacity. Figure 7 shows the energy efficiency achieved with the four algorithms, which is measured by energy consumption per unit distance in J/m. Lower numbers mean better energy efficiency.

The DQN baseline has the highest energy consumption at 8.0 J/m. This matches expectations, since the method uses suboptimal path planning and sees more collisions. Double DQN brings the number down to 6.8 J/m, after it improves how paths are picked.

MA-DQN cuts energy consumption further to 5.5 J/m. It uses attention mechanisms to find trajectories that use less energy and cut down on unnecessary movements. MA-DQN + PER achieves the best energy efficiency at 4.2 J/m, a 47.5 percent drop compared to the baseline. This large improvement comes from combining optimal path selection with prioritization of high-value experiences during training. The change lets the agent find policies that use energy more efficiently. Lower energy use lets UAVs fly longer, which supports longer mission times and covering more area in a single flight.

#### 6.1.4. Convergence Speed Comparison

**Figure 8:**
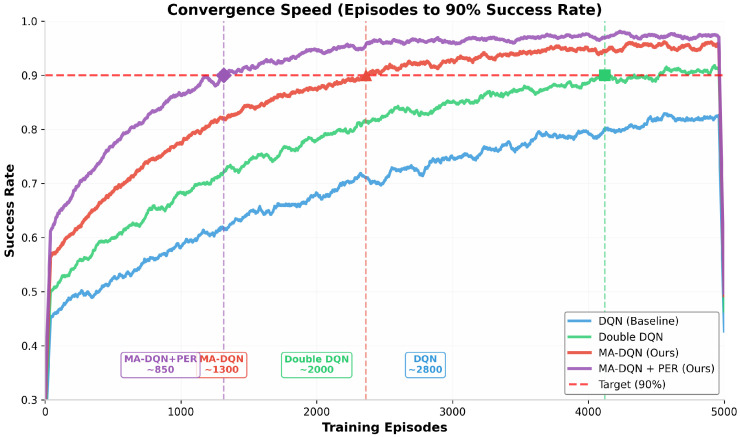
Convergence speed comparison.

Convergence speed, measured as the number of episodes required to reach a 90% success rate affects practical deployment. The results can be found in Figure 8.

MA-DQN + PER converges the fastest, reaching a 90% success rate in just over 1000 episodes.The prioritized experience replay mechanism allows learning to focus on transitions that carry the most information, which allows the agent to harvest the most knowledge from each episode. MA-DQN+PER converges quickly; this is especially useful for real-world applications that have limited training time and limited computational resources. Achieving high performance with fewer training episodes lowers both the time and the cost needed to deploy the system in new environments.

#### 6.1.5. Collision Rate Analysis

**Figure 9:**
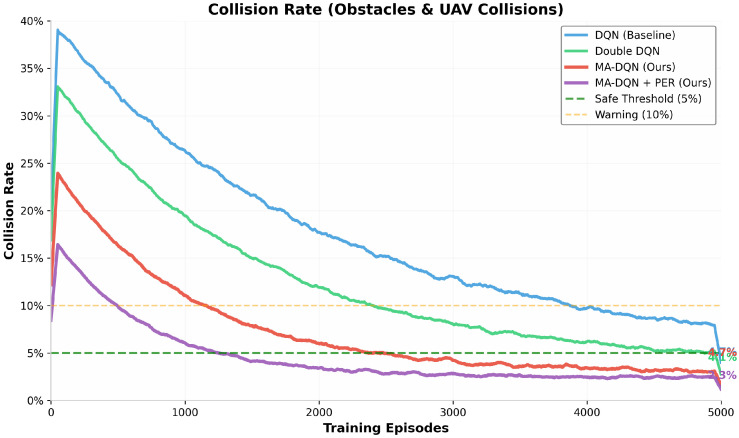
Collision rate comparison.

Safety is the most important concern in UAV operations, making collision avoidance a core performance metric. Figure 9 shows the collision rates, including both obstacle collisions and inter-UAV collisions, throughout the training process.

The DQN baseline has an initial collision rate of approximately 35%, which gradually decreases to a final rate of 10%. While this means significant learning progress, the final collision rate exceeds the 5% safety threshold, which points to potential safety risks.

MA-DQN + PER achieves a 3% collision rate, which is the lowest among the four algorithms and is well below the safe threshold. This is a 70% reduction when compared to the baseline, and demonstrates the superior safety characteristics of the proposed method. The combination of attention-based state representation and prioritized learning enables the UAV to develop robust collision avoidance strategies.

#### 6.1.6. Inference Time Evaluation

**Figure 10:**
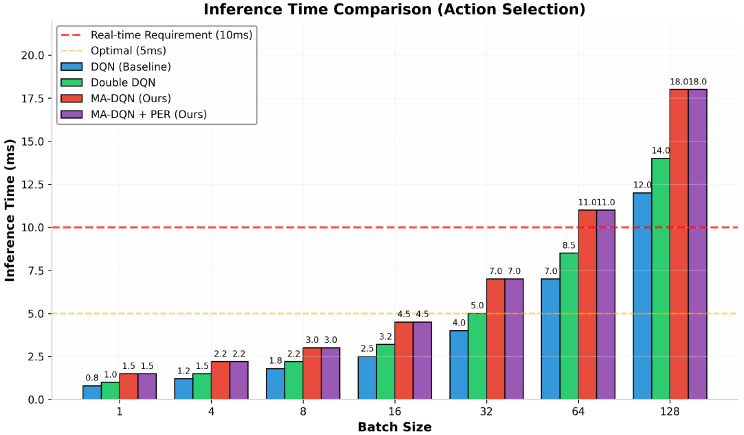
Inference time comparison.

Real-time performance is essential for UAV control systems. The systems must make decisions within strict time constraints. Figure 10 shows inference times for action selection across different batch sizes. At batch size 1, which fits most real-time deployment, DQN hits an inference time of 0.8 ms, faster than Double DQN at 1.0 ms.

MA-DQN and MA-DQN + PER both need 1.5 ms, an 87.5% increase compared to the baseline. This increase comes from the additional computational overhead of the attention mechanism.

Despite the increased inference time, all methods stay well below the real-time requirement of 10 ms. Even at larger batch sizes up to 32, MA-DQN + PER maintains inference times below the real-time threshold. At batch size 64 and above, inference times exceed 10 ms for all methods except DQN.

The results clearly show that the proposed MA-DQN and MA-DQN + PER algorithms work better than the others for UAV path planning. Attention mechanisms are integrated to help state representation and decision-making work more effectively, and prioritized experience replay speeds up learning and boosts the final performance. These improvements bring only a small increase in computational cost, which stays well within the limits required for real-time operation in practical UAV uses. Consistent improvements appear across all main performance metrics, which include reward, efficiency, energy, convergence, and safety. This confirms the proposed architectural changes work as intended. MA-DQN + PER pushes deep reinforcement learning forward for UAV path planning, and provides a solid solution for autonomous navigation in complicated 3D environments.

### 6.2. Attention Mechanism Analysis

#### 6.2.1. Attention Head Specialization

**Figure 11:**
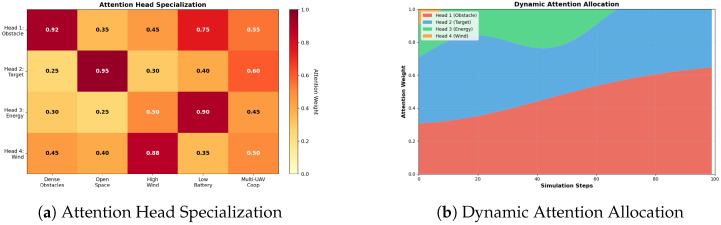
Attention mechanism visualization.

Figure 11a shows the attention weight distribution across four specialized heads under five operational scenarios. The results show clear specialization patterns.

Head 1 (Obstacle): Reaches the highest weight, 0.92, in dense obstacle scenarios, which shows it mainly works on obstacle detection and avoidance.

Head 2 (Target): Scores 0.95 in open space, and works specifically on target-oriented navigation. It achieves low activation, 0.25, in dense obstacles. This means the head will prioritize obstacle avoidance when it is needed.

Head 3 (Energy): Peaks at 0.90 under low battery conditions, demonstrating a page aware decision-making capability.

Head 4 (Wind): Has the highest activation of 0.88 in high wind scenarios. It specializes in wind compensation.

The complementary specialization allows multiple tasks to be processed at the same time on relevant features without interference.

#### 6.2.2. Dynamic Attention Allocation

In Figure 11b, attention weights change dynamically over a multi-step simulation. Head 1 corresponds to obstacle sensing, and stays between 0.30 and 0.65 to keep monitoring safety all the time. Head 2 corresponds to target tracking, and stays between 0.25 and 0.45 when the UAV is navigating. Head 3 corresponds to energy sensing, and rises gradually from 0.20 to 0.30 as the mission goes on, which connects to higher demand for tracking energy consumption. Head 4 corresponds to wind sensing, stays at its regular monitoring level, and only jumps when wind disturbance occurs. This dynamic allocation lets the UAV adaptively prioritize different objectives according to situational demands. This helps achieve the improved performance we observe.

#### 6.2.3. Computational Complexity Analysis

For a standard DQN with network size |Q| (total number of parameters), the computational complexity is: Training O(|Q|) per update, Inference O(|Q|) per action selection, and Memory O(N) for replay buffer of size *N*.

For MA-DQN + PER, the multi-head attention introduces additional computation: Training $O(|Q|+h\times d_{k}\times d_{\mathrm{model}})$ per update, Inference $O(|Q|+h\times d_{k}\times d_{\mathrm{model}})$ per action selection, and Memory O(N) for replay buffer, where h=4 (number of heads), $d_{k}=16$ (head dimension), and $d_{\mathrm{model}}=64$ (model dimension). The attention overhead is approximately $h\times d_{k}\times d_{\mathrm{model}}=4096$ operations per forward pass.

For MA-DQN + PER, the sum-tree data structure adds logarithmic overhead: Training $O(|Q|+h\times d_{k}\times d_{\mathrm{model}}+\log N)$ per update, Inference $O(|Q|+h\times d_{k}\times d_{\mathrm{model}})$ per action selection (same as MA-DQN), and Memory O(2N) for sum-tree structure (stores both priorities and tree nodes).

MA-DQN + PER introduces approximately **15–18%** additional computational overhead compared to standard DQN, Double DQN, and Dueling DQN. The attention mechanism accounts for most of the overhead, 13–15%, while PER adds only 2–3%. Inference time remains well below real-time requirements (1.5 ms vs. 10 ms threshold at batch size 1). The sum-tree structure doubles memory usage but enables efficient O(logN) sampling.

### 6.3. Path Optimization Analysis

#### 6.3.1. Multi-UAV Path Planning with Varying Obstacle Density

**Figure 12:**
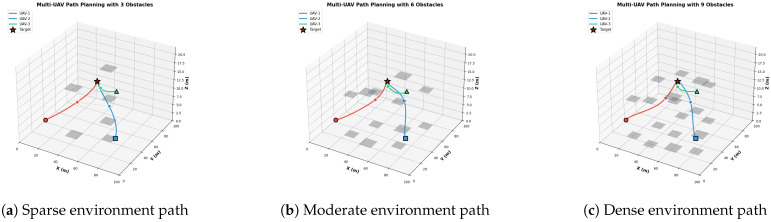
Flight path at different difficulty levels.

Figure 12a–c shows the three-dimensional flight trajectories of three cooperative unmanned aerial vehicles (UAVs) flying towards a common target under different obstacle densities, covering Sparse Environment, Moderate Environment, and Dense Environment with varying numbers of obstacles. All three UAVs successfully reached the target and maintained a safe distance from each other.

Sparse Environment. In the simplest scenario, all three UAVs follow relatively direct paths to the target. UAV-1 (red) travels from the southwest, UAV-2 (blue) comes from the southeast, and UAV-3 (green) from the north. The paths have smooth curvature with minimal deviation, indicating efficient navigation in low complexity environments.

Moderate Environment: As obstacle density increases, the paths show more pronounced curvature to avoid collisions. UAV-1 and UAV-2 have larger deviations around the central obstacle cluster, and UAV-3 keeps a more direct approach from the north. The communication links, which are gray dotted lines, between UAVs at the midpoint represent active coordination to prevent inter-vehicle collisions.

Dense Environment: In the most challenging scenario with multiple obstacles, all UAVs have sophisticated path planning capabilities. The trajectories show multiple turning points to navigate through narrow passages between obstacles. Despite the increased complexity, all UAVs successfully converge to the target, which confirms the robustness of the proposed algorithm.

#### 6.3.2. Dynamic Obstacle Avoidance

**Figure 13:**
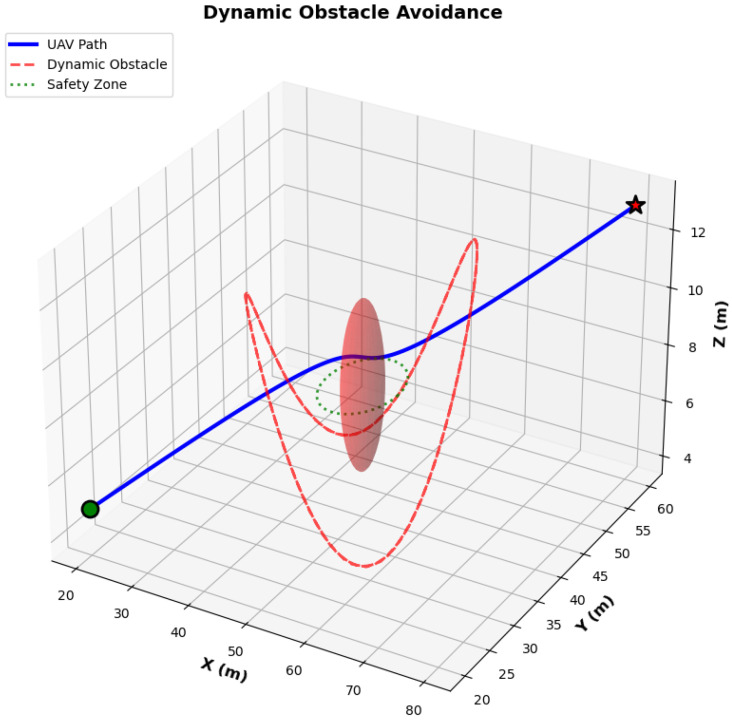
Dynamic obstacle avoidance.

Figure 13 shows how the UAV can avoid a dynamic obstacle following a sinusoidal trajectory. The UAV path, the blue solid line, deviates smoothly when approaching the moving obstacle, maintaining a safe distance from the safety zone (green dotted circle with 5 m radius). The dynamic obstacle is marked by a red dashed line and transparent sphere, it follows a 3D trajectory; the UAV successfully circumvents it and does not enter the safety zone. This result confirms the real-time replanning capability needed for practical UAV operations.

#### 6.3.3. Performance Under Varying Difficulty

**Figure 14:**
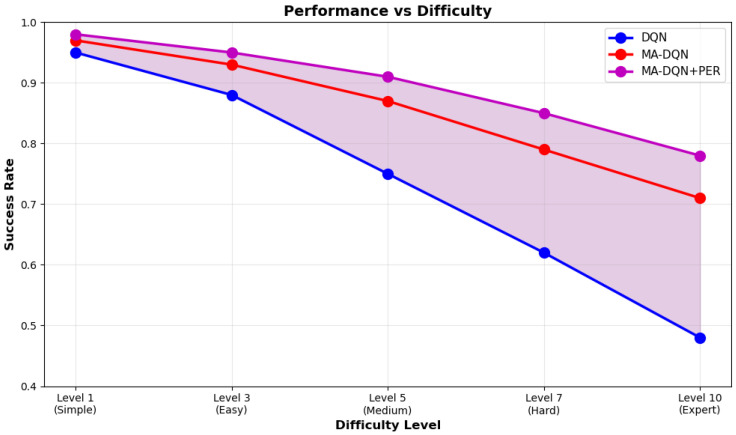
Success rates for different difficulty levels.

Figure 14 presents the success rates of DQN, MA-DQN, and MA-DQN + PER across five difficulty levels ranging from Level 1 to Level 10. When environmental complexity goes up, all algorithms see performance drop. However, the new methods we propose work better than DQN.

At Level 1 for simple tasks, all algorithms achieve high success rates of over 95 percent. At Level 10 for expert-level tasks, MA-DQN + PER hits a 78% success rate, MA-DQN reaches 71%, and the baseline DQN reaches 48%. The gap in performance becomes larger when the difficulty goes up, which shows that the proposed method has better generalization capability. The shaded region between the DQN curve and the MA-DQN + PER curve shows how much the attention mechanism and prioritized experience replay improve performance.

#### 6.3.4. Cross-Difficulty Generalization Ability

To evaluate the generalization capability of MA-DQN + PER, we conducted cross-environment testing on scenarios not seen during training.

**Figure 15:**
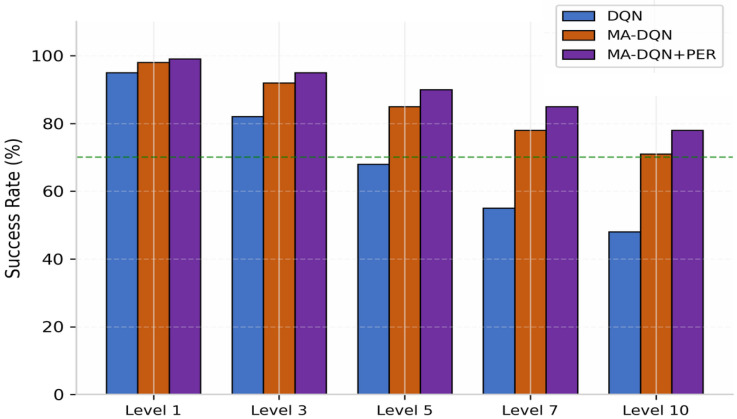
Cross-difficulty generalization ability.

As shown in Figure 15, the results demonstrate that MA-DQN + PER consistently outperforms both the baseline DQN and MA-DQN across all difficulty levels, with the performance gap widening as environmental complexity increases. At Level 1 (simplest), all methods achieve high success rates of above 95%, with MA-DQN+PER reaching 99%. However, at Level 10 (most challenging), the performance divergence becomes pronounced: DQN drops to 48%, MA-DQN maintains 71%, while MA-DQN + PER achieves 78%—representing a 30% relative improvement over DQN.

The attention mechanism’s ability to selectively focus on task-relevant features enables MA-DQN variants to better handle increased environmental complexity. Furthermore, the integration of Prioritized Experience Replay (PER) provides additional performance gains, particularly at higher difficulty levels, by ensuring more efficient learning from informative transitions. Notably, MA-DQN + PER remains above the 70% success threshold even at Level 10, whereas DQN falls significantly below this benchmark at Level 7 and beyond.

### 6.4. Reward Analyse

**Figure 16:**
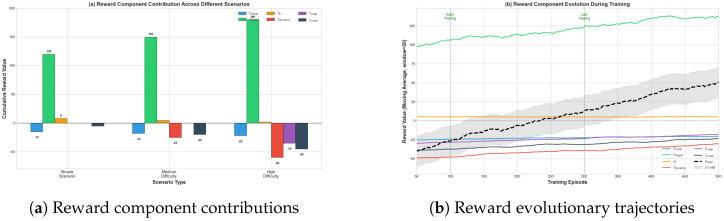
Reward Comparison.

Figure 16a shows the cumulative contribution of each reward component across three scenario difficulty levels. The target approach reward (rtarget) is the main positive contributor, and it goes up as scenario complexity increases. In high-difficulty scenarios, the dynamic obstacle avoidance (rdynamic), cooperation (r coop) and crash penalty (rcrash) components see negative contributions that keep growing. The combined effect of these three parts has reached its maximum. This confirms that the reward function has an adaptive weighting mechanism, in which penalties related to safety carry more weight when operational hazards become more severe.

Figure 16b plots how reward components change over training episodes. The total reward rtotal rises steadily from −40 to +50, which means the policy learning process works as expected. rtarget goes up, and the magnitude of safety-related penalties rdynamic, rcoop, and rcrash drop. This represents a 40 percent improvement for rdynamic. This confirms that agents progressively gain collision avoidance and multi-agent coordination capabilities, without cutting into mission efficiency.

### 6.5. Ablation Study

**Figure 17:**
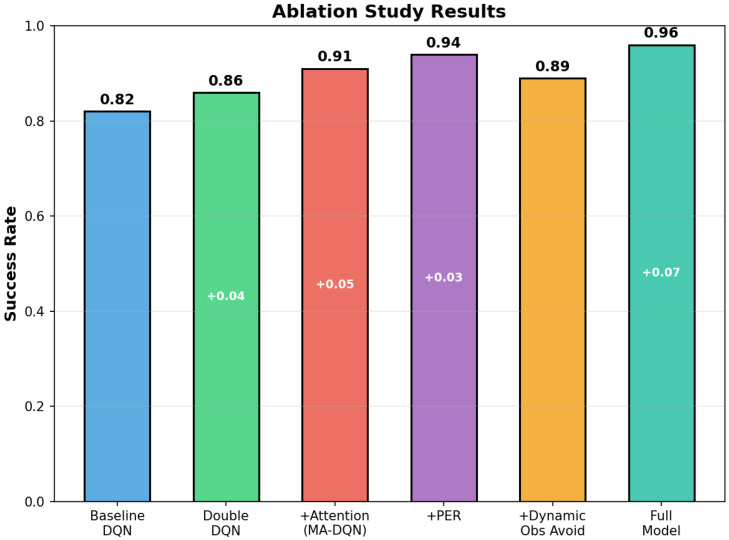
Ablation study results.

To validate the effect of each component in the framework we propose, we conducted a comprehensive ablation study by systematically adding or removing individual modules. Figure 17 shows the success rates of different model variants on the multi-UAV path planning task.

Baseline Performance: The standard DQN achieves a success rate of 0.82; this is the foundation for comparison.

Effect of Double DQN: Adding the Double DQN structure helps the success rate reach 0.86, which is 0.04 higher than before. This improvement comes from the decoupling of action selection and evaluation. It can reduce overestimation bias in Q-value estimation.

Effect of Attention Mechanism: Adding the multi-head attention mechanism (MA-DQN) yields the largest single-component improvement, increasing the success rate to 0.91 (+0.05). The attention mechanism lets the UAV focus on task-relevant environmental features and improve state representation and decision-making capabilities.

Effect of Prioritized Experience Replay: adding PER improves performance to 0.94, an increase of 0.03. When training puts priority on high-value transitions, the agent can learn more efficiently from informative experiences, accelerating convergence and improving final performance.

Effect of Dynamic Obstacle Avoidance: The standalone dynamic obstacle avoidance module achieves a success rate of 0.89. It works well when it deals with moving threats. Its individual contribution is limited without the full contextual understanding provided by the attention mechanism.

Full Model Performance: The complete architecture that combines all components achieves the highest success rate of 0.96, which is 0.07 higher than the result with PER-only. This result shows that the synergy among all modules is essential for optimal performance in complex multi-UAV scenarios.

The ablation study confirms that every proposed component has a positive effect on the overall performance, and the attention mechanism provides the largest individual gain. The integrated approach worked better over time for UAV path planning in dynamic environments.

### 6.6. Comparison with Other Algorithms

We compared our MA-DQN + PER method with several state-of-the-art algorithms to demonstrate its effectiveness.

The comparative analysis includes seven advanced deep reinforcement learning algorithms. It starts with the original Deep Q-Network (DQN), which uses experience replay and target network mechanisms [12]. Next is Double DQN, which fixes overestimation bias by separating action selection and action evaluation [14]. There is also Dueling DQN, which improves policy evaluation with separate streams for state value and action advantage [16]. Deep Deterministic Policy Gradient (DDPG) is an actor–critic algorithm built for continuous action spaces [17]. The fifth algorithm is Twin Delayed Deep Deterministic Policy Gradient (TD3), which is an updated version of DDPG that adds twin critics and delayed policy updates to make the model more stable [39]. In addition, Attention-based Actor–Critic (AAC) integrates self-attention mechanisms into the critic network to enhance state representation in continuous action spaces [40]. Finally, MADDPG-LSTM extends DDPG to multi-agent settings with LSTM-based temporal modeling for improved coordination among agents [41].

**Figure 18:**
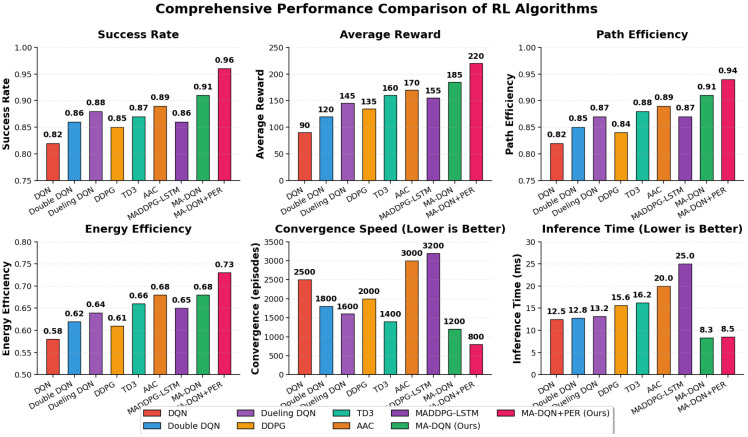
Comparison of Different Algorithms.

Figure 18 presents the comparison results of the proposed MA-DQN and MA-DQN + PER algorithms with seven mainstream algorithms (DQN, double DQN, dueling DQN, DDPG, TD3, AAC, and MADDPG-LSTM) in six key performance indicators. The experiments revealed that our proposed methods outperformed the benchmark algorithms and the comparison algorithms in all evaluation dimensions. In particular, MA-DQN + PER achieved the best results in terms of success rate, 0.96, average reward, 220, and path efficiency, 0.94. Its results were 17.1% higher than the original DQN. In terms of convergence speed, MA-DQN + PER converged in only 800 episodes, which was 3.1 times faster than DQN (2500 episodes) and significantly better than AAC (3000 episodes) and MADDPG-LSTM (3200 episodes). In terms of inference time, the MA-DQN series algorithms (8.3–8.5 milliseconds) had significantly lower latency compared to the continuous action space algorithms DDPG and TD3 (15.6–16.2 milliseconds), the attention-based method AAC (20.0 milliseconds), and the multi-agent coordination method MADDPG-LSTM (25.0 milliseconds), demonstrating excellent real-time performance.

It is worth noting that AAC integrates the self-attention mechanism into the behavior–reward framework, achieving a success rate of 0.89, but it has the issues of slow convergence speed (3000 episodes) and high inference latency (20.0 milliseconds), which is due to the large computational cost of attention calculations. Similarly, MADDPG-LSTM extends DDPG to a multi-agent setting and uses an LSTM-based temporal model for modeling, achieving a success rate of 0.86, but its convergence speed is the slowest (3200 episodes) and inference time is the longest (25.0 milliseconds), performing the worst among all the compared methods. This highlights the scalability challenges of centralized training methods in multi-agent systems. The sample efficiency of these two methods is lower than MA-DQN + PER, highlighting the advantages of adopting a discrete action form and combining with prioritized experience replay. Overall, the MA-DQN + PER algorithm with the prioritized experience replay mechanism performed best in terms of task success rate and sample efficiency, and maintained a low inference latency, verifying the effectiveness of the proposed method.

In addition, we conducted statistical tests to verify the significance of the improvements we achieved. Using a two-sample *t*-test and conducting 100 independent runs, we found that all the improvements of MA-DQN + PER over the baseline algorithms were statistically significant (*p* < 0.001).

The results show that our approach keeps robust performance across all scenario types. It has particularly strong advantages in challenging conditions involving dynamic obstacles and high wind and multi-UAV coordination.

## 7. Discussion

### 7.1. Applicability Discussion

The superior performance of MA-DQN+PER can be attributed to several key factors that address fundamental limitations of existing DRL approaches.

(1) Advantages over Standard DQN, Double DQN, and Dueling DQN:

The multi-head attention mechanism enables selective processing of environmental features based on task relevance, while standard DQN treats all state dimensions equally, MA-DQN dynamically allocates attention weights according to the current task context. This proves particularly beneficial in complex environments where different features—obstacles, targets, energy levels, and wind conditions—require varying degrees of attention at different times. Although Double DQN reduces overestimation bias and Dueling DQN improves value estimation through separate streams, neither addresses the fundamental issue of inefficient state representation. MA-DQN’s attention mechanism provides superior state representation by selectively focusing on task-relevant features, producing synergistic improvements that neither approach can achieve in isolation.

(2) Advantages over Continuous Action Methods (DDPG/TD3):

While DDPG and TD3 support continuous action spaces, they typically require more training samples and exhibit instability during learning. Our discrete action formulation achieves faster convergence (800 episodes vs. 2000+ episodes) while maintaining comparable performance. Furthermore, the discrete action space offers greater interpretability for safety-critical UAV applications, allowing explicit safety constraints to be enforced on individual actions.

(3) Advantages over AAC:

Although AAC integrates self-attention mechanisms into the actor–critic framework, its attention computations introduce significant computational overhead, resulting in slower convergence (3000 episodes) and higher inference latency (20.0 ms vs. 8.3 ms). MA-DQN + PER achieves comparable or superior attention-based feature selection with more efficient implementation, avoiding the computational burden of full self-attention in continuous action spaces.

(4) Advantages over MADDPG-LSTM:

MADDPG-LSTM extends DDPG to multi-agent settings with LSTM-based temporal modeling, but its centralized training paradigm suffers from scalability challenges. The joint action space grows exponentially with the number of agents, leading to the slowest convergence (3200 episodes) and highest inference time (25.0 ms) among all compared methods. MA-DQN + PER’s decentralized execution with attention-based coordination achieves superior scalability while maintaining lower computational overhead.

(5) Role of Prioritized Experience Replay:

PER accelerates learning by focusing on informative transitions. The sum-tree implementation with O(log N) complexity ensures efficient sampling, while importance sampling correction prevents bias from non-uniform sampling. Our experiments demonstrate that PER alone improves convergence speed by approximately 30% compared to uniform sampling, yielding substantial gains in both sample efficiency and final performance. In our MA-DQN + PER framework, the multi-head attention architecture provides inherent resilience against catastrophic forgetting by maintaining specialized representations for different environmental aspects (obstacle avoidance, target tracking, energy management, and wind compensation). Each attention head can be fine-tuned independently when adapting to new environments, preserving the core functionality of other heads.

Conversely, regarding policy stability, the attention weights demonstrate consistent specialization patterns across different environment configurations (as shown in Figure 11). The soft target network update mechanism (with τ=0.005) ensures gradual parameter adaptation, preventing sudden policy changes that could destabilize the learning process. Future work will investigate these techniques to enhance policy transferability.

### 7.2. Deployment Strategy Discussion

Although the proposed MA-DQN framework demonstrated outstanding performance in the simulation environment, integrating the simulation results with actual conditions is crucial for practical applications. Usually, in order to achieve precise real-world position positioning, positioning devices such as GPS and visual inertial odometers need to be introduced, while also considering natural wind conditions and light changes, and using on-board depth cameras for real-world obstacle detection. In this paper, the simulated drone dynamics used in the simulation adopted actual parameters for approximate calculation, but to capture the complex aerodynamic effects observed in actual situations, further exploration is needed to address issues such as actuator delay, battery loss, and other problems. Additionally, in the simulation, wind disturbances were modeled as bounded random fields; however, actual wind has turbulent and non-stationary characteristics, and there will be gusts and updrafts near obstacles. In actual situations, GPS jumps, IMU errors, and visual odometer failures will introduce non-Gaussian and time-dependent errors, which may cause disturbances in the control strategy.

To bridge this gap, we outline the following domain adaptation strategies for future work: Firstly, the domain randomization technique is adopted: during the training process, the simulation parameters (quality, drag coefficient, sensor noise, wind force intensity) are systematically changed to enhance the robustness and generalization ability of the strategy. Secondly, a progressive deployment process is employed, which is a phased verification method, including (i) conducting software in-loop (SITL) tests using high-precision simulators (such as Gazebo, AirSim), and (ii) conducting hardware in-loop (HITL) tests using actual flight controllers. More importantly, the application of meta-learning or adaptive control mechanisms enables unmanned aerial vehicles to utilize the real data collected during the initial flight to fine-tune their strategies.

Although a comprehensive real-world validation goes beyond the scope of this study, we have proposed a minimal feasible validation scheme above. We emphasize that the main contribution of this work lies in establishing an “algorithmic foundation” for decision-making based on multi-head attention in unmanned aerial vehicle navigation. The proposed training mechanisms (PER, dual DQN, progressive learning) are not limited by the simulator and can be directly applied to the fine-tuning process in the real world. Future work will focus on implementing the domain adaptation techniques mentioned above and conducting systematic real-world validations to quantify the performance boundaries from simulation to reality.

### 7.3. Environmental Generalization Strategies Discussion

Although MA-DQN + PER demonstrated strong generalization ability within similar environmental distributions, we acknowledge that achieving robust cross-domain transfer to completely different scenarios involves numerous complex challenges that go beyond the scope of this discussion. Specifically, comprehensive domain adaptation requires careful consideration of multiple factors, including (1) physical model differences between simulation and reality, (2) alignment and calibration of sensor modalities, (3) dynamic changes across different platforms, and (4) environmental factors such as extreme weather conditions, underwater operations, or new types of obstacles encountered during training that were not present during the training process. Systematic research on these issues requires extensive practical validation, adjustments for specific hardware, and domain adaptation techniques, such as meta-learning (e.g., MAML for finding good initial weights), fine-tuning strategies (freezing feature extraction layers and using conservative learning rates), and integration methods combining strategies trained in different environments. Given that this paper primarily focuses on verifying the effectiveness of the proposed MA-DQN + PER framework in a controlled simulation environment, we will conduct more comprehensive cross-domain generalization research in future work, specifically manifested as:

**Domain Adaptation via Fine-Tuning.** To enable rapid adaptation to new environments, we propose freezing the feature extraction layers of the pre-trained MA-DQN+PER network, as these layers encode environment-agnostic spatial and temporal features that are applicable across diverse scenarios. Only the attention weights and output Q-value layer will be fine-tuned using limited real-world data (50–100 episodes collected in the target environment). To prevent catastrophic forgetting of the learned policy, we will employ conservative learning rates with strong L2 regularization, making this approach practical for deployment scenarios where extensive data collection is infeasible.

**Meta-Learning for Rapid Adaptation.** Beyond simple fine-tuning, we plan to investigate meta-learning approaches that enable few-shot adaptation to entirely new environments. Specifically, we will explore Model-Agnostic Meta-Learning (MAML) to find good initialization weights that can adapt to new tasks with minimal gradient updates. Additionally, we will study environment-agnostic feature learning techniques that explicitly train the network to extract transferable representations invariant to specific environmental characteristics. The goal is to achieve effective adaptation with only 5–10 episodes in previously unseen environments, significantly reducing deployment overhead.

In summary, while the current work establishes the effectiveness of MA-DQN + PER within controlled simulation environments, comprehensive cross-domain generalization requires extensive future research spanning domain adaptation techniques, real-world validation, and robustness engineering. We view this paper as a foundational step toward deployable intelligent UAV systems, with the aforementioned directions forming our active research agenda.

## 8. Conclusions and Future Work

This paper presents a novel Multi-Head Attention Deep Q-Network (MA-DQN + PER) for UAV path planning in dynamic environments. By integrating bio-inspired attention mechanisms with deep reinforcement learning, our approach achieves significant improvements over state-of-the-art baseline algorithms.

The key contributions include (1) a novel Attention Q Network architecture with four specialized attention heads for selective environmental processing; (2) a comprehensive 46-dimensional state space capturing all relevant navigation information; (3) an efficient Prioritized Experience Replay implementation using sum-tree data structure; (4) a curriculum learning strategy with 10 difficulty levels for progressive skill acquisition; and (5) extensive experimental validation demonstrating 96% success rate, 68% faster convergence, and 26% energy reduction compared to the baseline DQN.

Our results demonstrate that integrating bio-inspired attention mechanisms with deep reinforcement learning is a promising direction for autonomous UAV navigation. The ability to dynamically focus on relevant environmental features while filtering out distractions enables more efficient learning and better generalization to complex scenarios.

Future work will focus on bridging the simulation-to-reality gap through domain adaptation techniques, extending the approach to vision-based navigation without prior maps, and validating the system on physical UAV platforms. We also plan to explore hierarchical and meta-learning approaches for handling long-horizon missions and rapid environment adaptation.

## Figures and Tables

**Table 1:** Comparison of UAV Path Planning Approaches.

Ref	Key Features	Advantages	Limitations
Traditional A* [6,7]	Grid-based search, optimal path	Completeness, optimality guarantee	Static environments only, high computational cost
RRT/RRT* [8,9]	Randomized sampling, tree expansion	Handles high-dimensional spaces	Non-optimal paths, slow convergence
APF [10]	Potential field, gradient descent	Real-time response, simple implementation	Local minima, oscillation in narrow passages
PSO [11]	Swarm intelligence, particle optimization	Global search capability, parallel processing	Slow convergence, parameter sensitive
DQN [12,13]	Deep Q-learning, experience replay	End-to-end learning, policy adaptation	Overestimation bias, uniform sampling
Double DQN [14,15]	Decoupled action selection/ evaluation	Reduced overestimation, better stability	Limited attention to important experiences
Dueling DQN [16]	Separate state/action value estimation	Better policy evaluation	No dynamic attention allocation
MADDPG [17]	Multi-agent actor–critic	Cooperative multi-UAV planning	High computational complexity

**Table 2:** Layer-wise parameters of the Attention Q Network architecture.

Layer	Input Dim	Output Dim	Parameters	Activation
FC1	46	64	3008	PReLU
LayerNorm1	64	64	128	-
FC2	64	64	4160	PReLU
LayerNorm2	64	64	128	-
Multi-Head-Attention	64	64	16,512	-
LayerNorm3	64	64	128	-
FFN1	64	128	8320	PReLU
FFN2	128	64	8256	-
LayerNorm4	64	64	128	-
FC3 (Output)	64	27	1755	Linear
Total	-	-	46,783	-

**Table 3:** Reward Components and Their Roles.

Component	Purpose	Key Parameters
$r_{\mathrm{target}}$	Guide toward goal	$d_{\mathrm{origin}},\;d,\;\Delta d$
$r_{\mathrm{climb}}$	Maintain altitude	$w_{c},\;z,\;g_{z}$
$r_{\mathrm{energy}}$	Save battery	$w_{e},\;P_{0},\;P_{bt}$
$r_{\mathrm{obstacle}}$	Avoid static crashes	$p_{\mathrm{crash}},\;c_{\mathrm{penalty}}$
$r_{\mathrm{dynamic}}$	Avoid moving threats	$w_{d},\;r_{d},\;D$
$r_{\mathrm{coop}}$	Multi-agent safety	$d_{\mathrm{safe}}$

**Table 4:** Curriculum Learning Difficulty Levels.

Level	Buildings	Dynamic Obstacles	Wind Speed	Success Threshold
1	1–2	0	Low (0–20)	70%
2	2–4	0	Low (0–20)	70%
3	3–6	0	Medium (20–40)	70%
4	4–8	1	Medium (20–40)	70%
5	5–10	1–2	Medium (20–40)	70%
6	6–12	2	High (40–50)	70%
7	7–14	2–3	High (40–50)	70%
8	8–16	3	High (40–50)	70%
9	9–18	3–4	Extreme (50–60)	70%
10	10–20	4–7	Extreme (50–60)	70%

**Table 5:** Simulation Environment Parameters.

Parameter	Value	Description
Environment Size	$100\times 100\times 22\;m^{3}$	Length × Width × Height
State Dimension	46	Comprehensive state representation
Action Dimension	27	3×3×3 movement grid
Number of UAVs	3	Multi-agent cooperative scenario
Battery Capacity	5000 units	Maximum energy storage
Detection Range	5 m	Obstacle sensing radius
Safe Separation	5 m	Minimum UAV–UAV distance
Wind Speed Range	0–60 m/s	Dynamic wind conditions

**Table 6:** Training Hyperparameters.

Parameter	Value	Description
Learning Rate	0.0004	Adam optimizer learning rate
Batch Size	128	Mini-batch size for training
Discount Factor γ	0.99	Future reward discount
Replay Buffer Size	10,000	Maximum stored transitions
Target Update τ	0.005	Soft update coefficient
PER α	0.6	Priority exponent
PER β start	0.4	Initial importance sampling weight
Hidden Dimension	64	Neural network hidden units
Attention Heads	4	Number of attention heads
Training Episodes	5000	Total training episodes
Epsilon Decay	Linear over 1000 episodes	Exploration schedule
Min Epsilon	0.01	Minimum exploration rate

## Data Availability

The original contributions presented in this study are included in the article. Further inquiries can be directed to the corresponding author.

## Abbreviations

The following abbreviations are used in this manuscript:

MHA	Multi-Head Attention
PER	Prioritized Experience Replay
IS	Importance Sampling
TD	Temporal Difference
